# Mitochondrial Dynamin-Related Protein Drp1: a New Player in Cardio-oncology

**DOI:** 10.1007/s11912-022-01333-w

**Published:** 2022-10-01

**Authors:** Yali Deng, Doan T. M. Ngo, Jessica K. Holien, Jarmon G. Lees, Shiang Y. Lim

**Affiliations:** 1 Department of Surgery and Medicine, University of Melbourne, Melbourne, Victoria Australia; 2 O’Brien Institute Department, St Vincent’s Institute of Medical Research, Fitzroy, Victoria Australia; 3 School of Biomedical Science and Pharmacy, College of Health, Medicine and Wellbeing, Hunter Medical Research Institute & University of Newcastle, New Lambton Heights, New South Wales Australia; 4 School of Science, STEM College, RMIT University, Melbourne, Victoria Australia; 5 Drug Discovery Biology, Faculty of Pharmacy and Pharmaceutical Sciences, Monash University, Melbourne, Victoria Australia; 6 National Heart Research Institute Singapore, National Heart Centre, Singapore, Singapore

**Keywords:** Dynamin-related protein, Mitochondria, Doxorubicin, Cardiomyopathy, Cancer

## Abstract

**Purpose of Review:**

This study is aimed at reviewing the recent progress in Drp1 inhibition as a novel approach for reducing doxorubicin-induced cardiotoxicity and for improving cancer treatment.

**Recent Findings:**

Anthracyclines (e.g. doxorubicin) are one of the most common and effective chemotherapeutic agents to treat a variety of cancers. However, the clinical usage of doxorubicin has been hampered by its severe cardiotoxic side effects leading to heart failure. Mitochondrial dysfunction is one of the major aetiologies of doxorubicin-induced cardiotoxicity. The morphology of mitochondria is highly dynamic, governed by two opposing processes known as fusion and fission, collectively known as mitochondrial dynamics. An imbalance in mitochondrial dynamics is often reported in tumourigenesis which can lead to adaptive and acquired resistance to chemotherapy. Drp1 is a key mitochondrial fission regulator, and emerging evidence has demonstrated that Drp1-mediated mitochondrial fission is upregulated in both cancer cells to their survival advantage and injured heart tissue in the setting of doxorubicin-induced cardiotoxicity.

**Summary:**

Effective treatment to prevent and mitigate doxorubicin-induced cardiotoxicity is currently not available. Recent advances in cardio-oncology have highlighted that Drp1 inhibition holds great potential as a targeted mitochondrial therapy for doxorubicin-induced cardiotoxicity.

## Introduction

The global cancer burden is projected to reach 28.4 million cases in 2040, a 47% rise from 2020 [1]. With recent medical advancements in early detection and effective cancer therapies, the number of long-term cancer survivors is estimated to surpass 22.1 million by 2030 in the USA [2]. The 10-year survival rate of the twenty most common malignancies sits at approximately 50% and is greater than 80% in melanoma, lymphoma, breast and uterine cancers in developed countries [3]. Paradoxically, the improved prognosis for cancer patients is accompanied by a growing population with secondary cardiac complications. Doxorubicin, which belongs to a class of antibiotic anthracyclines, is a potent cytotoxic chemotherapeutic drug widely prescribed for cancer treatment [4]. Doxorubicin is commonly used to treat children and adults with solid tumours (e.g. breast and ovarian cancer) and haematological malignancies (e.g. leukaemia and lymphoma) [5]. Unfortunately, the clinical usage of doxorubicin is associated with dose-dependent and cumulative cardiotoxicity that may lead to congestive heart failure [6]. It is estimated that half of the children diagnosed with cancer receive anthracyclines as part of their cancer therapy [7]. Of note, the prevalence of doxorubicin-induced cardiac dysfunction has reached 30% of adult survivors of childhood cancer in western populations [8, 9].

Although the advent of more targeted cancer therapies has reduced the usage of doxorubicin as the first-line treatment, doxorubicin remains the mainstay treatment against cancers that are refractory to those new treatment and have no druggable targets [10, 11]. Doxorubicin exerts its anti-tumour effects predominately through DNA damage, disruption of mitochondrial function and production of reactive oxygen species (ROS), which ultimately cause cell death [12–15]. Despite its effective anti-tumour effects, the clinical utility of doxorubicin has been restricted by its tendency to cause cardiovascular complications including left ventricular systolic dysfunction and heart failure [6, 16]. The incidence of acute doxorubicin-induced cardiotoxicity is about 11%, usually occurring within days to months after administration [17]. Common acute manifestations include arrhythmia and left ventricular dysfunction [6, 18, 19]. Chronic doxorubicin-induced cardiomyopathy usually occurs within a year, with an overall incidence of 9%, but it may also manifest in cancer survivors up to 30 years after their last treatment regimen. Chronic doxorubicin-induced cardiomyopathy is progressive and irreversible, characterised by a significant reduction in left ventricular fractional shortening and ejection fraction, which may lead to heart failure [17, 20–22]. Notably, the risk of developing congestive heart failure is primarily related to the cumulative dose, increasing from 5% when the dose of doxorubicin is 400 mg/m^2^ to 26% at 550 mg/m^2^ and 48% when the dose exceeds 700 mg/m^2^ [23]. Devastatingly, patients who develop congestive heart failure often have a poor prognosis, with a 2-year mortality rate of about 50% [24].

The difference in the time of onset of doxorubicin-induced cardiomyopathy may be attributed to the genetic polymorphisms of individuals that have altered antioxidant capacity, membrane permeability and metabolisms, which render certain individuals more susceptible to cardiac damage. For example, a genetic disorder known as hemochromatosis that causes iron overload is likely to increase the susceptibility of an individual to doxorubicin-induced cardiotoxicity [25]. Additionally, the basal mitochondrial capacity and mitochondrial DNA fitness in cardiomyocytes may also determine the severity of cardiac dysfunction and their resistance to doxorubicin-induced cardiomyopathy [26–28]. Other risk factors that may lend predilection for doxorubicin-induced cardiomyopathy include those that are treatment associated (concomitant cardiotoxic therapies such as monoclonal antibody trastuzumab and mediastinal radiotherapy) and patient related (sex, age, existing chronic conditions such as cardiovascular disease, hypertension, liver disease and diabetes mellitus) [29–31].

The molecular mechanisms underlying doxorubicin-induced cardiotoxicity are complicated and multifaceted, as reviewed extensively elsewhere [32]. Despite extensive research over the past decades which has suggested its putative mechanisms, the precise molecular signalling pathways associated with the cardiotoxic effects of doxorubicin remain elusive [32]. The majority of studies have concluded cardiomyocyte death as the principal mechanism [33]. The main causes of cardiomyocyte death include death receptor activation, oxidative stress, calcium dysregulation, mitochondrial dysfunction and DNA damage [31, 34–37].

## Current Strategies to Treat Doxorubicin-Induced Cardiotoxicity

Many pharmacotherapies have been investigated for their potential to protect the heart against doxorubicin-induced cardiotoxicity. Unfortunately, effective strategies to prevent or remediate doxorubicin-induced cardiomyopathy are yet to be established in clinical practice [38]. It is evident that the anti-cancer effects of doxorubicin overlap with its cardiotoxic effects; hence, there is a possibility that cardioprotective agents could also reduce the oncological efficacy of doxorubicin by acting through the same mechanisms.

The current clinical practice guideline for patients with established cardiomyopathy and heart failure includes pharmacotherapies with angiotensin-converting enzyme (ACE) inhibitors, angiotensin receptor blockers, β-adrenergic blockers, combined angiotensin receptor and neprilysin inhibitors, mineralocorticoid receptor antagonists and sodium-glucose cotransporter 2 inhibitors [39, 40]. Although these medications were not developed specifically for doxorubicin-induced cardiomyopathy, some have been proven to be effective for heart failure due to doxorubicin and have been clinically implemented as a standard guideline-directed medical therapy for patients who have developed cardiomyopathy and heart failure after doxorubicin treatment [39, 40]. For example, co-administration of ACE inhibitors such as enalapril with doxorubicin has been shown to reduce doxorubicin-induced cardiac dysfunction through the preservation of mitochondrial respiratory efficiency and reduction in free radical production [41, 42]. Treatment with non-selective β-adrenergic blockers such as propranolol and carvedilol has also shown to improve cardiac function and reduce cardiac injury in the setting of doxorubicin-induced cardiotoxicity [43–47]. Despite these positive findings, there is limited evidence available regarding their cardioprotective mechanisms in the setting of doxorubicin-induced cardiomyopathy and whether these protective agents might interfere with the anti-cancer effects of doxorubicin.

Other agents targeting mitochondrial processes such as antioxidants have also shown to exert cardioprotective effects by detoxifying excess ROS and reducing apoptosis in a pre-clinical study [48]. Yet the cardioprotective effect of antioxidants did not translate into clinical benefits in terms of preventing or reversing cardiac dysfunction and heart failure in patients receiving doxorubicin [49, 50]. To date, the only Food and Drug Administration–approved cardioprotective drug against doxorubicin-induced cardiotoxicity is dexrazoxane, an iron-chelator with an additional ability to inhibit topoisomerase II-beta in cardiomyocytes [51]. However, the clinical use of dexrazoxane is limited to patients with advanced breast cancer who are receiving doxorubicin at a cumulative dose of over 300 mg/m^2^ due to the risk of developing myelosuppression and secondary tumours that could interfere with the anti-cancer activity of doxorubicin [4, 52, 53]. It is important to note that recent randomised controlled trials reported that dexrazoxane did not compromise anti-tumour efficacy of doxorubicin or increase the risk of developing secondary tumours in children and adolescents [54, 55].

Given the limited success of existing therapeutic agents in preventing or treating doxorubicin-induced cardiotoxicity, finding other approaches that could effectively mitigate cardiotoxicity without compromising the effectiveness of doxorubicin is an important unmet medical need.

## Mitochondrial Dynamics

**Fig. 1:**
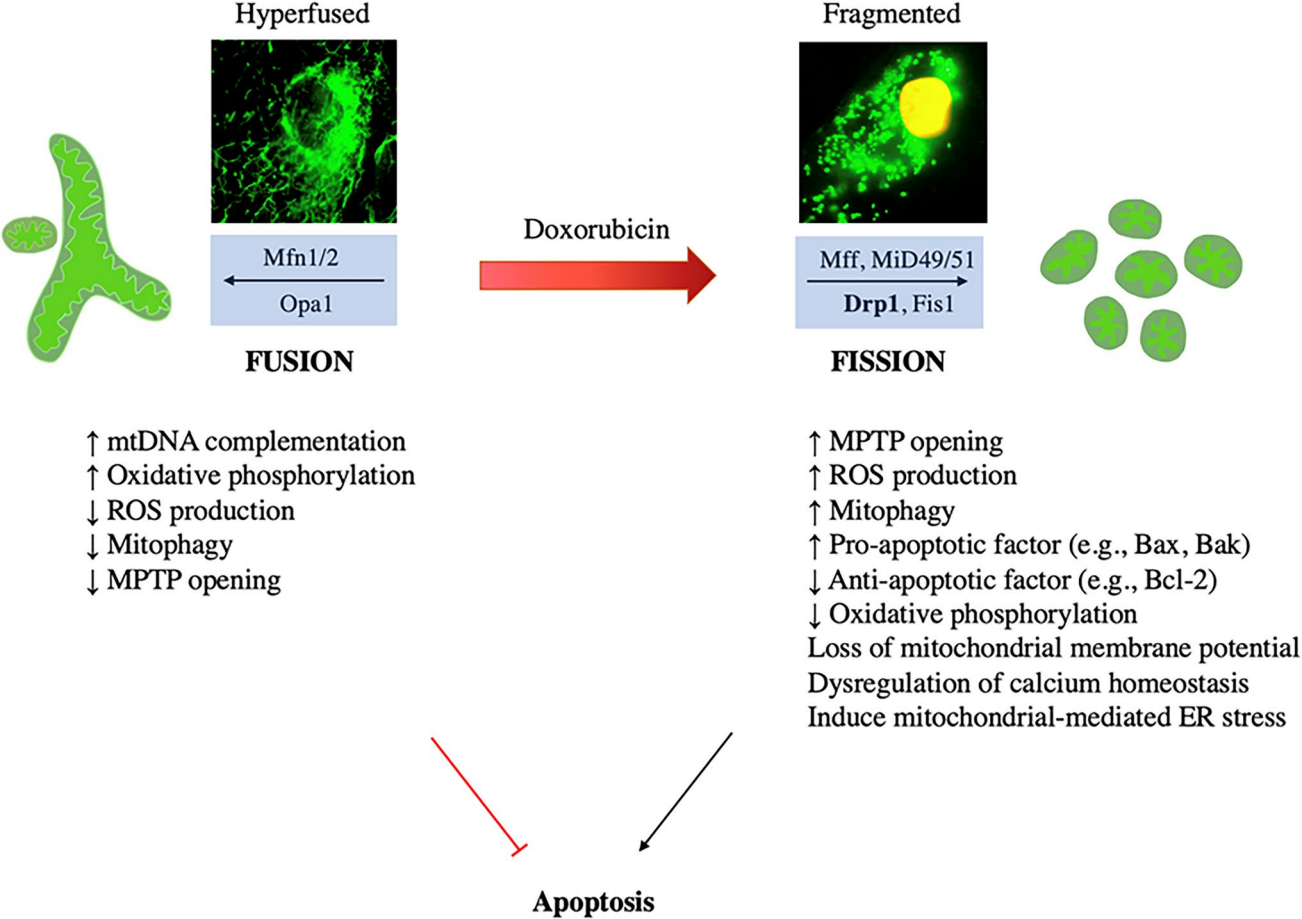
The role of Drp1 upregulation in doxorubicin-induced cardiotoxicity. Mitochondrial morphology is governed by fusion and fission proteins, which in turn affects mitochondrial functions. Pro-fusion (Mfn1&2, Opa1) and pro-fission (Drp1, Fis1, Mff and Mid49/51) proteins are core mediators in fine-tuning mitochondrial morphology and function. Perturbation in the fission and fusion processes can shift the mitochondrial dynamics towards mitochondrial fragmentation (fission) or mitochondrial elongation and formation of a hyper-fused network (fusion). Doxorubicin upregulates Drp1 expression which tilts the mitochondrial dynamic balance towards fission to trigger various downstream effects that ultimately lead to cardiomyocyte apoptosis and cardiac injury. The Drp1 inhibitor is a promising pharmacological agent that inhibits the excessive doxorubicin-mediated mitochondrial fission and attenuates its cardiotoxic effects. Mfn1 mitofusin 1, Mfn2 mitofusin 2, Opa1 optic atrophy 1, Drp1 dynamin-related protein 1, Fis1 mitochondrial fission protein 1, Mff mitochondrial fission factor, Mid49 mitochondrial dynamics protein 49 kDa, Mid51 mitochondrial dynamics protein 51 kDa, DOX doxorubicin, mtDNA mitochondrial deoxyribonucleic acid, MPTP mitochondrial permeability transition pore, ROS reactive oxygen species

Mitochondria are a major cellular target of doxorubicin. Mitochondrial damage is a prominent feature of doxorubicin-induced apoptosis. Mitochondrial fusion and fission proteins are known to play an important role in regulating mitochondrial morphology and function such as mitochondrial metabolism and bioenergetics, intracellular ROS production and calcium signalling [56]. Mitochondria exist in a spectrum of shapes and sizes, ranging from small fragments of spheres or rods (fission) to elongated tubules and interconnected networks (fusion) [57]. Mitochondrial fusion and fission processes are tightly regulated by a group of highly conserved dynamin superfamilies known as large GTPases, recognised for their intracellular membrane remodelling activities through their self-assembly and GTP hydrolysing capabilities [58]. These mechanochemical GTPase proteins include mitofusin 1 (Mfn1), mitofusin 2 (Mfn2) and optic atrophy 1 (Opa1), which enhance mitochondrial fusion. In contrast, mitochondrial fission is mediated by dynamin-related protein 1 (Drp1), mitochondrial fission 1 protein (Fis1), mitochondrial fission factor (Mff) and mitochondrial dynamic protein of 49 and 51 kDa (Mid49/51) in mammals [57, 59] (Fig. 1).

A concerted balance of mitochondrial fission and fusion is important for preserving mitochondrial morphology, which in turn influences the physiological functions of a cell. Mitochondrial fission participates in (1) inheritance and compartmentalization of mitochondria during cellular division [60], (2) removal of defective mitochondria by mitophagy [61], (3) intracellular redistribution of mitochondria via a cytoskeletal-mediated transmission [62], (4) regulation of calcium homeostasis [63], (5) programmed cell death through the release of pro-apoptotic factors [64] and (6) G2/M cell cycle progression during mitosis [65].

On the other hand, mitochondrial fusion is an important process to maintain a healthy mitochondrial population by preventing the accumulation of defective mitochondria in a cell population. Mitochondrial fusion defends cells from the deleterious effects of mitochondrial deoxyribonucleic acid (DNA) mutations by enabling the exchange of genetic content with impaired mitochondria through a process known as functional complementation [66, 67]. Moreover, fused mitochondrial networks are required for maximal ATP synthesis by stimulating oxidative phosphorylation in energetically active cells or when cells respond to stress stimuli such as nutrient deprivation to preserve energy [68, 69].

## The Role of Drp1 in Cancer

Dysregulation of mitochondrial dynamics is often linked to cancer tumourigenesis and progression, as reviewed comprehensively elsewhere [70, 71]. Specifically, the upregulation of mitochondrial Drp1 with concomitant downregulation of Mfn2 has been shown to promote tumourigenesis in many cancers including lung, gastric, breast, glioma, colon, ovarian, pancreatic and melanoma [72]. In most instances, Drp1-mediated mitochondrial fission promotes replication, invasion and migration, and drug tolerance in tumour cells thereby enhancing tumour growth [73]. Thus, inhibition of Drp1 has been reported to exert anti-tumour effects shown to enhance apoptosis and reduce proliferation of tumour cells [74–77].

A putative small molecule inhibitor of Drp1, Mdivi-1, has been widely employed to study the pathophysiological roles of Drp1-mediated mitochondrial fission. Mdivi-1 is a quinazolinone derivative and a reversible allosteric inhibitor of Drp1 discovered from a chemical library in a yeast-based assay [78]. By inhibiting the GTPase activity of Drp1, Mdivi-1 has been found to reduce Drp1 self-assembly on the mitochondrial outer membranes and mitochondrial fission in mammalian cells [78, 79]. Mdivi-1 exerts cytotoxic effects in hyper-proliferative cancer cells by inducing death receptor–mediated apoptosis and inhibiting cell cycle progression [80–82]. In breast cancer cells, Mdivi-1 significantly reduced metabolic reprogramming, mitophagy and cell viability [83]. Similarly, short interference ribonucleic acid (siRNA)–mediated Drp1 knockdown or pharmacological inhibition of Drp1 using Mdivi-1 reduced breast cancer cell metastasis and invasiveness [76, 84].

A major clinical challenge in cancer therapy is addressing chemoresistance, which promotes cancer recurrence and metastasis and hampers the clinical outcomes of cancer patients. Drp1 inhibition, either via gene silencing of Drp1 or pharmacologically using Mdivi-1, has been shown to sensitise multiple cancer cell lines to the cytotoxic effects of chemotherapeutic agents. For example, Mdivi-1 or siRNA against Drp1 enhanced cisplatin-induced apoptosis in breast carcinoma (MDA-MB-231), renal cancer cells (Caki-1), lung carcinoma (A549 and A1299), colon carcinoma (HCT116) and ovarian carcinoma (SKOV3, PA1 and A2780) [80, 85, 86]. Similarly, treatment with Mdivi-1 has been shown to enhance cisplatin-induced apoptosis in hepatocellular carcinomas by suppressing Drp1-mediated mitophagy [87]. Drp1 inhibition also sensitised malignant melanoma, lung cancer and osteosarcoma cells to tumour necrosis factor–related apoptosis-inducing ligand (TRAIL)–mediated apoptosis through a caspase-dependent pathway [88]. Courtois and colleagues reported that Mdivi-1 can chemosensitise pancreatic ductal adenocarcinomas to the chemotherapeutic agent gemcitabine by inhibiting mitochondrial fission and inducing mitochondrial dysfunction that led to the accumulation of defective mitochondria and ultimately cell death [89]. Treatment with Mdivi-1 has also been demonstrated to augment Taxol cytotoxicity in breast carcinoma MDA-MB-231 cells by inducing spindle abnormalities [90].

In contrast, some studies have reported a pro-tumourigenic effect of Drp1 inhibition. Li et al. showed that suppression of mitochondrial translocation of Drp1 through its phosphorylation of Drp1 at serine residue 637 promoted hepatocellular cancer cell metabolic reprogramming and survival [91]. In another study, inhibition of Drp1-dependent mitochondrial fission via siRNA against Drp1 or Mdivi-1 compromised the cytotoxic effect of cisplatin in MDA-MB-231 breast cancer cells via an inhibition of cisplatin-induced intracellular ROS production and recovery of mitochondrial membrane potential [92]. Furthermore, Tang and colleagues have demonstrated that IR-783, a heptamethine cyanine dye commonly used for imaging cancer cells, exhibits anti-cancer properties by increasing Drp1-mediated mitochondrial fission in MDA-MB-231 breast cancers [93]. Knockdown of Drp1 markedly blocked IR-783-induced mitochondrial fission, loss of mitochondrial membrane potential, ATP depletion, mitochondrial permeability transition pore opening and apoptosis. Additionally, IR-783 also induced apoptosis and inhibited tumour growth in MDA-MB-231 in vivo in a xenograft model [93]. More recently, silibinin, a bioactive natural polyphenolic flavonoid, has been shown to suppress cervical cancer cell proliferation by inducing G2/M cell cycle arrest and apoptosis via Drp1-dependent mitochondrial fission both in vitro and in vivo*.* The same study also reported that knockdown of Drp1 reversed the anti-cancer effects of silibinin-induced cell cycle arrest by inhibiting the mitochondrial fission pathway [94].

These contradictory findings of Drp1 inhibition to act as both pro-apoptotic and anti-apoptotic events in cancers cells may be attributed to the differences in the study model employed, chemotherapeutic agents used, treatment regimens, duration of experiment, types of apoptotic stimuli applied, signalling pathways being investigated, metabolic activity of tumours and/or stage of tumourigenesis. To illustrate, in breast carcinoma MDA-MB-231 cells, Mdivi-1 enhanced the cytotoxic effect of cisplatin when cells were cultured under normoxic conditions [86] but attenuated the cytotoxic effect of cisplatin when cells were cultured under hypoxic conditions [92].

## The Role of Drp1 in Doxorubicin-Induced Cardiotoxicity

Mitochondria provide more than 90% of the ATP required by cardiomyocytes to support normal cardiac contractility, and mitochondrial fusion and fission proteins play critical roles in cardiac homeostasis [95]. Doxorubicin promotes cardiomyocyte apoptosis by altering mitochondrial structure and function, which is associated with a disruption in mitochondrial redox homeostasis and dysregulated mitochondrial dynamics. Mechanistically, doxorubicin induces excessive mitochondrial ROS production, disrupts mitochondrial membrane structure and depolarises the mitochondrial membrane potential, leading to cell death [96]. Moreover, doxorubicin has been shown to cause excessive mitochondrial fragmentation in cardiomyocytes through the increased phosphorylation of Drp1 at serine 616 and decreased expression of the Opa1 protein, which accelerates mitochondria-dependent apoptosis [97]. This positions mitochondrial fusion and fission proteins as promising therapeutic targets for protection against doxorubicin-induced cardiotoxicity by restoring the balance of mitochondrial dynamics [98] (Fig. 1).

Recent studies have also suggested a possible role of endoplasmic reticulum stress in exacerbating the cardiotoxic effects of doxorubicin. Bagchi and colleagues have shown that doxorubicin-induced oxidative stress activated the opening of the mitochondrial permeability transition pore and increased pro-apoptotic Bax protein expression in primary rat cardiomyocytes [99]. This in turn enhanced the protein expression of endoplasmic reticulum chaperone proteins and DNA damage-inducible transcriptor-3 in the endoplasmic reticulum, leading to endoplasmic reticulum stress and apoptosis [99]. Notably, Drp1-mediated fission also plays a role in ER stress by promoting remodelling of endoplasmic reticulum microdomains to constrict mitochondria and promote/drive mitochondrial fission [100]. Pharmacological Drp1 inhibition using Mdivi-1 effectively suppresses endoplasmic reticulum remodelling–driven mitochondrial fission and autophagy in cardiometabolic proteostasis [100]. Altogether, targeting dysregulated Drp1-dependent mitochondrial fission may provide a novel approach to reduce doxorubicin-induced cardiotoxicity. Indeed, pre-clinical studies have demonstrated the cardioprotective effect of Drp1 inhibition in doxorubicin-induced cardiotoxicity in both in vitro and in vivo settings (Table 1).

**Table 1:** The role of Drp1 in anthracycline-induced cardiotoxicity

Approaches	Study models	Outcomes
siRNA of Drp1 [101]	Neonatal rat cardiomyocytes treated with 2 µM of doxorubicin for 12 h	Reduced doxorubicin-induced mitochondrial fragmentation, cytochrome c release, loss of mitochondrial membrane potential and cell death
siRNA of Drp1 [102]	H9c2 cardiac myoblast cells treated with 750 nM of doxorubicin for 24 h	Attenuated doxorubicin-induced mitochondrial fragmentation, mitophagy flux and cell death
	Ventricular cardiomyocytes isolated from adult mice treated with 3 µM of doxorubicin for 24 h	Reduced doxorubicin-induced mitochondrial fragmentation
Drp1 knockout mice [102]	Drp1 heterozygous knockout mice received a single dose of doxorubicin (15 mg/kg) via intraperitoneal injection for 3 days	Protected against doxorubicin-induced cardiac injury. Reduced doxorubicin-induced mitochondrial fragmentation, oxidative stress and lipid peroxidation
Mdivi-1 [103]	Ex vivo rat heart subjected to acute ischaemia–reperfusion injury and treated with 1 µM doxorubicin ± 1 µM of Mdivi-1, given at reperfusion for 2 h	Protected against doxorubicin-induced myocardial dysfunction and infarction
	Primary adult rat cardiomyocytes treated with 1 µM of doxorubicin ± 1 µM of Mdivi-1 for 4 h	Reduced doxorubicin-induced mitochondrial depolarisation and hyper-contracture of cardiomyocytes
Mdivi-1 [104]	H9c2 rat cardiomyoblasts pre-treated with 1 µM of Mdivi-1 for 30 min and then incubated with 5 µM of doxorubicin for 24 h	Reduced doxorubicin-induced mitochondrial fragmentation and cell death
Mdivi-1 [105]	Neonatal rat ventricular cardiomyocytes were pre-treated with 1 µM of Mdivi-1 for 30 min ± 5 µM of doxorubicin for 24 h	Prevented doxorubicin-induced mitochondrial fission and apoptosis
Mdivi-1 [106]	H9c2 cardiomyocytes treated with 1 µM of doxorubicin ± Mdivi-1*	Attenuated doxorubicin-induced apoptosis, upregulation of Drp1 expression and autophagic activities

*siRNA* short interference ribonucleic acid, *Mdivi-1* mitochondrial division inhibitor 1, *Drp1* dynamin-related protein 1

^*^Concentration of Mdivi-1 was not reported in the study

A recent study by Catanzaro and colleagues has demonstrated that Drp1 knockdown attenuated doxorubicin-induced cardiac injury in H9c2 cardiomyoblasts and mice in vivo, which was associated with reduced doxorubicin-induced mitochondrial fission and cell death [102]. This finding is in keeping with an earlier study that reported a cardioprotective effect of siRNA-mediated Drp1 knockdown in neonatal rat cardiomyocytes subjected to doxorubicin-induced toxicity [101]. Using a pharmacological approach, Gharanei and colleagues have demonstrated that inhibition of Drp1 with Mdivi-1 exerted a cardioprotective effect in Langendorff-perfused rat hearts subjected to doxorubicin-induced cardiac injury by reducing mitochondrial fission and cardiomyocyte apoptosis [103]. Similarly, Mdivi-1 effectively reduced doxorubicin-induced cardiomyocyte apoptosis, mitochondrial fission and autophagic activities in H9c2 cardiomyocytes [104–106]. Mechanistically, the cardioprotective effect of Mdivi-1 has been attributed to an improvement in mitochondrial function through restoring the mitochondrial membrane potential, reducing mitochondrial ROS production, lowering cytosolic calcium overload and delaying hyper-contracture of cardiomyocytes [103, 107–109].

In addition to the direct approach of targeting Drp1, various studies have indirectly targeted Drp1 by pharmacologically inhibiting upstream signalling pathways to demonstrate the role of Drp1-mediated mitochondrial fission in doxorubicin-induced cardiomyopathy. For example, treatment with LCZ696, an angiotensin receptor-neprilysin inhibitor and widely known as sacubitril-valsartan, reduced doxorubicin-induced cardiotoxicity by improving cardiac function and reduced cardiomyocyte apoptosis in vivo, partly via Drp1-mediated mitochondrial fission [104]. In H9c2 cells, sacubitril-valsartan attenuated doxorubicin-induced cardiomyocyte contractile dysfunction and blunted the increase in Drp1-mediated mitochondrial fission [104]. Using the mitophagy inhibitor liensinine, Liang et al. reported a reduction in doxorubicin-induced cardiomyocyte apoptosis, cardiomyocyte hypertrophy and cardiac contractile dysfunction in mice receiving concurrent treatment of liensinine [110]. Importantly, the cardioprotective effect of liensinine was mediated by the Rab7-Erk-Drp1 signalling axis [110]. Liensinine maintained cell survival via decreasing Rab7 level which led to a reduction in ERK and Drp1 protein phosphorylation levels. Consequently, liensinine reversed mitochondrial fragmentation and ultimately inhibited mitochondrial fission–mediated cell death.

The traditional Chinese herbal medicine, Shenmai, has also been used to demonstrate cardioprotection against doxorubicin-induced cardiotoxicity via maintaining mitochondrial homeostasis. Treatment with Shenmai suppressed excessive doxorubicin-induced mitochondrial fission by increasing the ratio of fusion protein L-Opa1 to S-Opa1 and phosphorylation of Drp1 at serine 637 residue. Consequently, treatment with Shenmai rescued H9c2 cardiomyoblasts from doxorubicin-induced apoptosis, excessive mitochondrial ROS production and loss of mitochondrial membrane potential [97]. More recently, Klotho, an anti-ageing protein, was found to suppress doxorubicin-induced apoptosis in neonatal rat ventricular cardiomyocytes and H9c2 cardiomyoblasts. In doxorubicin-treated mice, Klotho effectively reduced cardiac cell death and improved cardiac function through attenuation of Drp1 Ser616 phosphorylation [105]. In addition, a neuraminidase 1 inhibitor called oseltamivir has also been reported to exert cardioprotective effects against doxorubicin-induced cardiotoxicity via suppressing Drp1-dependent mitophagy*.* In vivo, oseltamivir improved doxorubicin-induced cardiac dysfunction in male Sprague–Dawley rats. In vitro, oseltamivir suppressed myocardial apoptosis through inhibition of Drp1-mediated excessive fission and mitophagy in both doxorubicin-treated rats and H9c2 cardiomyoblasts [106]. Taken together, various pharmacological approaches have underscored the importance of Drp1 inhibition as a potential mechanism to protect cardiomyocytes against doxorubicin-induced cardiotoxicity.

## Conclusion and Future Perspectives

One of the key challenges when choosing a cardioprotective agent to prevent or mitigate doxorubicin-induced cardiotoxicity is the parallel evaluation of the anti-cancer activity of doxorubicin to ensure that its oncological efficacy is not compromised. Notably, Drp1-mediated mitochondrial fission activity is upregulated in most cancer cells conferring a survival advantage and in heart tissue as a key contributor to doxorubicin-induced cardiotoxicity. Current pre-clinical animal and cell culture studies have suggested that Drp1 inhibition is a promising approach for protecting the heart against doxorubicin-induced injury without compromising the anti-cancer properties of doxorubicin in treating various types of cancer. Moreover, Drp1 inhibition appears to exert anti-cancer effects in some cancer cells [70, 71] and might also have synergistic anti-cancer effects with doxorubicin and other chemotherapeutic agents in cancers where dysregulation of mitochondrial dynamics occurs; however, this remains to be determined.

To date, the cardioprotective effects of Drp1 inhibition in doxorubicin-induced cardiotoxicity have only been demonstrated in limited pre-clinical studies using either an in vivo animal model or in vitro primary rodent cardiomyocytes and H9c2 rat cardiomyoblasts (Table 1). With the advent of stem cell technology, human cardiomyocytes derived from induced pluripotent stem cells (iPSC) generated from individual somatic cells represent a superior pre-clinical human model for disease modelling and development of personalised treatments. Indeed, many studies have successfully employed human iPSC–derived cardiomyocytes to model doxorubicin-induced cardiotoxicity [111–115].

Cardiomyocyte death is well known as the primary cause of doxorubicin-induced cardiomyopathy [116, 117]. Since the myocardium is a heterocellular tissue comprised of both cardiomyocytes and non-cardiomyocytes such as cardiac fibroblasts, endothelial cells, smooth muscle cells and autonomic neurons, there is a potential involvement of non-cardiomyocytes in doxorubicin-induced cardiotoxicity. Indeed, recent studies have reported non-cardiomyocytes as cytotoxic targets of doxorubicin [118]. Increased oxidative stress triggered by doxorubicin mediates a plethora of cellular signalling pathways including cardiac hypertrophy, fibrosis, impaired angiogenesis, cell senescence and death [118]. Cardiac fibrosis is triggered by necrotic and apoptotic cell damage as well as pathological remodelling in response to excessive ROS production. Studies have reported a role for doxorubicin-induced cardiotoxicity in triggering apoptosis, a pro-fibrotic phenotype and senescence in mouse and rat cardiac fibroblasts [119–121]. Additionally, endothelial cells are also implicated in doxorubicin-induced oxidative stress [122, 123], which is correlated with endothelial cell apoptosis and reduced endothelial plasticity [124]. Treatment with doxorubicin has also been shown to promote premature senescence in smooth muscle cells isolated from the human aorta, characterised by excessive ROS generation, abnormal morphology, accumulation of DNA damage foci and increased activity of senescence associated-β-galactosidase [125]. Given the complexity of the underlying pathogenesis of doxorubicin-induced cardiomyopathy, a better model such as the use of a 3-dimensional multicellular cardiac organoid consisting of both cardiomyocytes and non-cardiomyocytes is needed to better dissect the mechanisms underlying doxorubicin-induced cardiomyopathy [126]. In addition, disease models that better recapitulate the cellular heterogeneity of the native heart will help to further elucidate mechanisms underlying the cardioprotective effects of various pharmacotherapies and facilitate novel drug development.

Pharmacological inhibition of Drp1 has been demonstrated to be effective at attenuating doxorubicin-induced cardiotoxicity by reducing cardiomyocyte death in pre-clinical animal and cell culture studies (Table 1). Among all Drp1 inhibitors, Mdivi-1 is the most extensively studied Drp1 inhibitor in pre-clinical settings. However, recent evidence has reported off-target effects of Mdivi-1, including the inhibition of mitochondrial complex I [127]. While Mdivi-1 may continue to be a useful research tool, its clinical utility will be restricted due to its unfavourable off-target effects, low potency and poor water solubility [78, 109]. Other available Drp1 inhibitors include P110 (peptide inhibitor that inhibits the interaction between Drp1 and Fis1) and Dynasore (a non-specific inhibitor of dynamins). P110 improved cell function and mitochondrial dynamics in pre-clinical models of myocardial ischemia–reperfusion injury and neurodegenerative diseases [128, 129]. Dynasore has shown to promote apoptosis and reduce cell proliferation in A549 lung cancer cells [130, 131]. On the other hand, Dynasore exerted cardioprotective effects by improving left ventricular end-diastolic pressure and increasing the survival rate of cardiomyocytes upon ischemia–reperfusion injury in mice [132].

While existing Drp1 inhibitors have exhibited promising therapeutic effects in pre-clinical models, evidence supporting their specificity and selectivity to the human Drp1 GTPase binding site is lacking. The cardio-oncology field requires better tool compounds to understand the therapeutic potential of Drp1-mediated fission inhibition in doxorubicin-induced cardiomyopathy. Recently, two novel Drp1 inhibitors, Drpitor and Drpitor1a, have been discovered to be more potent and specific than Mdivi-1 in inhibiting the GTPase activity of Drp1 [133]. Drpitor1 and Drpitor1a have also displayed anti-cancer effects by reducing cell proliferation and inducing apoptosis in lung cancer cells while conferring cardioprotective effects against ischemia–reperfusion injury in rodent models [133]. Further studies are required to validate the effectiveness of Drpitors in inhibiting human Drp1 GTPase and its therapeutic potential in pre-clinical models. Importantly, the benefits of Drp1 inhibition in humans are currently unknown, and future clinical trials are needed to evaluate the safety and efficacy of Drp1 inhibitors in clinical settings. In summary, Drp1 inhibition holds potential in translating a targeted mitochondrial therapy for doxorubicin-induced cardiotoxicity into clinical reality.
